# Effectiveness of a formulation containing peptides and vitamin C in treating signs of facial ageing: three clinical studies

**DOI:** 10.1111/ics.12665

**Published:** 2020-12-22

**Authors:** S. Escobar, A. Valois, M. Nielsen, B. Closs, D. Kerob

**Affiliations:** ^1^ BAAS Institute Buenos Aires Argentina; ^2^ L’Oréal Research & Innovation Chevilly Larue France; ^3^ Laboratoires Vichy Levallois Perret France; ^4^ Silab Saint‐Viance France

**Keywords:** formulation, peptides, vitamin C, anti‐ageing, skin physiology/structure, skin barrier

## Abstract

**Objective:**

Vitamin C and peptides are widely used in cosmetic products but there is a paucity of clinical studies showing that the formulations are effective in treating signs of facial ageing. These 3 clinical studies evaluated the effectiveness of an anti‐ageing formula containing natural vitamin C (10%), biopeptides (rice and lupin), hyaluronic acid, and Vichy volcanic mineralising water, in amber glass ampoules with no preservatives (Peptide‐C ampoules).

**Methods:**

Dansyl chloride fluorescence labelling compared cell turnover for Peptide‐C ampoules vs untreated skin in 32 female subjects. Study 2, an open clinical study, evaluated the efficacy on wrinkles of Peptide‐C ampoules by investigator clinical scoring based on Dynamical Atlas visual assessment (N = 40) and subject self‐assessment questionnaires (N = 47). Study 3, an open clinical study, evaluated wrinkles by instrumental quantification with 3D fringe projection analysis (N = 40) and subject questionnaires (N = 51).

**Results:**

The mean cell turnover was faster for skin treated with Peptide‐C ampoules compared to untreated skin (17.1 days vs. 19.2 days; *P* < 0.0001). In study 2, after 28 days application of Peptide‐C ampoules, clinical grading of crow’s‐feet wrinkles, forehead wrinkles and nasolabial folds decreased by 9%, 11% and 5%, respectively (all *P* < 0.05 vs baseline). Of 47 subjects, 77%, 64% and 79% indicated their skin seemed smoothed out, fine lines were less visible, and skin complexion was more radiant, respectively. In study 3, the number of wrinkles decreased by 11.5% after 29 days application of Peptide‐C ampoules vs baseline (*P* < 0.05) and 65% of subjects responded the fine lines were less visible.

**Conclusion:**

This formulation of a combination of anti‐ageing ingredients in ampoules, allowing a minimalist formula, showed significant results on improving facial wrinkles and radiance.

## Graphical abstract

A formulation containing peptides and vitamin C, in innovative packaging allowing a minimalist formula, demonstrated effectiveness and high subject satisfaction for wrinkle reduction and skin rejuvenation.

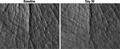

## Résumé

**Objectif:**

La vitamine C et les peptides sont régulièrement utilisés dans les produits dermocosmétiques mais il existe peu d’études cliniques sur l’efficacité des formulations sur les signes du vieillissement cutané du visage. Trois études cliniques ont évalué l’efficacité d’une formule anti‐âge contenant de la vitamine C naturelle (10%), des biopeptides (riz et lupin), de l’acide hyaluronique et de l’eau minéralisante volcanique de Vichy, dans un format d’ampoules en verre ambré, sans conservateur (ampoules Peptide‐C).

**Méthodes:**

Une première étude a comparé par la technique de chlorure de Dansyl le renouvellement cellulaire avec la formulation ampoules Peptide‐C et la peau non traitée chez 32 sujets féminins. La seconde étude, en ouvert, a évalué l’efficacité clinique sur les rides des ampoules Peptide‐C en se reposant sur les Atlas Dynamiques (N=40) et les questionnaires d’auto‐évaluation des sujets (N=47). La troisième étude, ouverte, a évalué les rides par quantification instrumentale avec l’analyse de projection de franges 3D (N=40) et les questionnaires d’autoévaluation des sujets (N=51).

**Résultats:**

Le renouvellement cellulaire était plus rapide pour la peau traitée avec des ampoules de Peptide‐C comparées à la peau non traitée (17.1 jours contre 19.2 jours ; p<.0001).

Dans l’étude 2, après 28 jours d’application des ampoules Peptide‐C, l’évaluation clinique des rides de la patte d’oie, du front et des plis naso‐labiaux a montré une amélioration de 9 %, 11 % et 5 %, respectivement (tous p<0,05 vs baseline). Sur 47 sujets, 77%, 64% et 79% ont indiqué que leur peau semblait respectivement lissée, que les ridules étaient moins visibles et que le teint de la peau était plus radieux.

Dans l’étude 3, le nombre de rides a diminué de 11,5 % après 29 jours d’application des ampoules Peptide‐C par rapport à la baseline (p<0,05) et 65 % des sujets ont répondu que les ridules étaient moins visibles.

**Conclusion:**

Cette combinaison d’ingrédients anti‐âge dans un format d’ampoules, et une formulation minimaliste, a montré des résultats significatifs sur l’amélioration des rides faciales et de l’éclat du teint

## Introduction

Increasing longevity and the desire for maintaining a youthful appearance is stimulating demand for products that help to reduce the signs of ageing skin, especially wrinkles, lack of firmness of cutaneous tissues (ptosis), vascular disorders and uneven pigmentation [1]. Being directly in contact with the external environment, such as solar radiation and air pollution, the skin is where the first visible signs of ageing occur. All exposures (internal biological factors and external environmental factors) to which an individual is subjected to from conception to death, known as the exposome, have an impact on the pathophysiology of skin ageing and oxidation plays a major role [2, 3].

Vitamin C is a well‐known ingredient for its antioxidant and anti‐ageing properties [4]. A study in superoxide dismutase 1 (Sod1)‐deficient mice demonstrated that combined treatment with collagen peptide and vitamin C attenuated age‐related skin atrophy by reducing oxidative damage [5]. Normal skin contains high concentrations of vitamin C, which supports important functions, such as collagen synthesis and antioxidant protection against UV‐induced photodamage. Vitamin C has been attributed to depigmenting and brightening skin due to its ability to inhibit tyrosinase, thus suppressing melanin formation [6].

Numerous biologically active peptides with specific activities in the human body have been discovered in the last 50 years and have been used in cosmetic products as effective anti‐ageing ingredients [7, 8]. Polypeptides or oligopeptides are composed of amino acids and can imitate peptides sequence in molecules such as collagen or elastin. They act as messenger molecules in the body, or when synthetic peptides are topically applied via a cosmetic product, and migrate to target cells where they stimulate the production of collagen, rebuilding the dermal matrix [9]. Anti‐ageing peptides may also stimulate elastin and lumican synthesis to reduce the appearance of wrinkles and to increase firmness [10, 11, 12].

The biological peptide complex in the formulation evaluated in our studies contains a peptide hydrolysate of di‐ and tripeptides from rice and lupin protein that have been optimised for rapid assimilation by skin cells and passage through the skin barrier [13]. Previous *in vitro* studies have reported that di‐ and tripeptides derived from rice stimulate the proliferation of fibroblasts and stimulate messenger RNA expression of pro‐collagen, collagen VII and fibrillin‐1 [13].

Although peptides and vitamin C are established anti‐ageing ingredients, the formulation is critical to maintain active and stable antioxidants. Vitamin C formulated at low pH has been shown to improve its bioavailability in the stratum corneum [14]. Formulations of vitamin C (present as 15% L‐ascorbic acid) at pH levels between 2 and 5 were topically applied to pig skin, and tissue levels of L‐ascorbic acid were enhanced only at formulation pH levels below 3.5 [14]. The formulation described here has been developed at low pH and contains pure vitamin C (10%), peptides (rice and lupin), hyaluronic acid and Vichy volcanic mineralising water (LiftActiv Specialist Peptide‐C ampoules, Vichy, Paris, France [Peptide‐C ampoules]). The aim of these 3 clinical studies was to evaluate, by investigator and biometrological assessments, as well as subject self‐assessment, the effectiveness of Peptide‐C ampoules topical formulation on wrinkles and radiance.

## Methods

### Study 1: investigator‐blinded study with dansyl chloride cell proliferation testing

In this investigator‐blinded study, 35 female subjects aged 36–64 years old applied Peptide‐C ampoules (12 drops twice daily) to one designated forearm, determined by a computer‐generated randomisation schedule, for 2 weeks pre‐treatment and 3 weeks treatment. The evaluator was blinded to treatment designation to the right or left forearm. A patch measuring approximately 3.5 cm × 3.5 cm and containing 0.2 g of dansyl chloride (5% in petrolatum) fluorescent dye was placed on each forearm volar surface for approximately 24 h before removal. Subjects were instructed to continue application of Peptide‐C ampoules to the designated forearm for 3 weeks while the contralateral forearm remained untreated. During this 3‐week treatment period, remaining fluorescence was measured daily on each site (treated and untreated) using a Wood’s Light.

### Study 2: open study with investigator clinical scoring and subject questionnaires

A total of 51 female subjects aged 40–60 years old were selected for this open study. Inclusion criteria included having crow’s‐feet wrinkles of grade ≥ 2 and < 4 (on a scale from 0 [no visible crow’s‐feet wrinkles] to 6 [very visible crow’s‐feet wrinkles]); forehead wrinkles of grade ≥ 2 (on a scale from 0 [no visible forehead wrinkles] to 5 [very visible forehead wrinkles]); and a nasolabial fold of grade ≥ 3 (on a scale from 0 [no visible nasolabial fold] to 5 [very visible nasolabial fold]). The Peptide‐C ampoules formulation was applied on the face and neck for 28 days (half an ampoule twice daily). Investigator (dermatologist) clinical scoring based on Dynamical Atlas (Atlas Morphing, L'Oréal) visual assessment was performed on the whole face on day 0 and day 28 in 40 women, and subject self‐assessment questionnaires were completed by 47 subjects on day 0 (after application) and day 28.

### Study 3: open study with instrumental quantification and subject questionnaires

In this second open study, 53 female subjects aged 40–55 years old were enrolled to apply Peptide‐C ampoules on the face and neck for 29 days (half an ampoule twice daily). The main inclusion criteria specifically included having crow’s‐feet wrinkles of grade ≥ 2 and < 5 (on a scale from 0 [no visible crow’s‐feet wrinkles] to 6 [very visible crow’s‐feet wrinkles]) with a main crow’s‐foot wrinkle of at least 2 cm in length that was not crossed by any other wrinkles. The side where the print of the crow’s‐foot wrinkle was performed was designated by computer‐generated randomisation. The efficacy on wrinkles was assessed by instrumental quantification on 40 women using software (Quantirides) and 3D fringe projection analysis (Toposurf 3D) on day 1 (before application of Peptide‐C ampoules) and day 30. Subject self‐assessment questionnaires were completed by 51 women on day 1 (immediately after Peptide‐C ampoules application) and day 30.

### Treatment formulation

The anti‐ageing formula contains a high concentration of pure vitamin C (10%), peptides (rice and lupin), hyaluronic acid and Vichy volcanic mineralising water packaged in daily‐dose, amber glass ampoules requiring no preservatives (Peptide‐C ampoules).

### Ethical considerations

Clinical studies were conducted according to ICH CPMP Good Clinical Practices guidelines and the Declaration of Helsinki (1964, and its subsequent modifications). All subjects gave written informed consent.

### Statistical analysis

Comparisons between both groups (treated and untreated sites or day 28/day 29 vs baseline results) were performed by paired Student’s t‐test if normal distribution (checked by Shapiro–Wilk test) or by Wilcoxon signed ranks test if the Shapiro–Wilk normality test was inferior to 1%. Comparisons between treated and untreated sites, or between results from day 28/day 29 vs baseline, were performed by two‐sided significance test at the 5% level.

## Results

### Study 1: dansyl chloride testing

Of 35 subjects enrolled, 32 (91%) completed 3 weeks treatment with dansyl chloride testing; two subjects discontinued for personal reasons and one due to an adverse event (AE; moderate erythema and itching on the treatment side, which was considered as probably related to the investigational product).

The mean cumulative fluorescence score at day 22 was lower for skin treated with Peptide‐C ampoules compared to untreated skin (59.6 vs. 64.9; *P* < 0.0001) and the mean area under the curve was lower at 56.5 vs. 61.7, respectively (*P* < 0.0001). Mean cell turnover, defined as the number of days required for disappearance of the fluorescent dye, was faster for treated skin (17.1 days) compared to untreated skin (19.2 days; *P* < 0.0001).

### Study 2: investigator clinical scoring

Of 51 subjects selected, 47 completed the study and clinical grading was performed on 40 subjects. Reasons for discontinuation were 2 subjects withdrew consent, one protocol deviation and one subject discontinued due to an AE of mild erythema from day 1 with stinging/burning from day 13; the AE was likely related to the investigational product and the subject discontinued the study.

All 47 subjects were Caucasian females with mean age 52.6 ± 4.8 years old (range: 40–60 years). At baseline, 22 subjects (47%) had sensitive skin and most had Fitzpatrick skin phototype II (47%) or III (47%) and 6% had phototype I.

**Figure 1:**
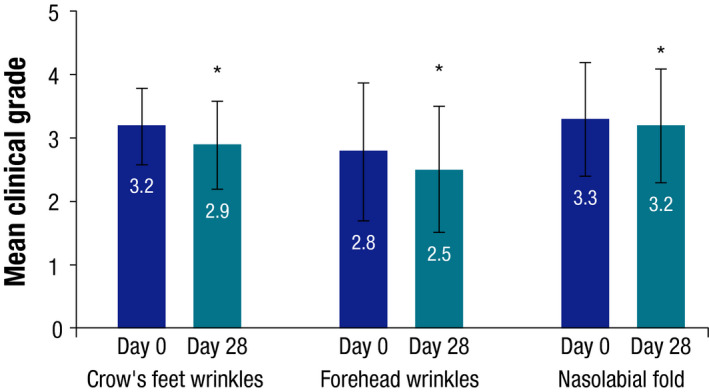
Mean (±SD) clinical grades at baseline and day 28 for crow’s‐feet wrinkles, forehead wrinkles and nasolabial fold (N = 40) (* all *P* < 0.05 compared to baseline).

On day 28, mean clinical grades of crow’s‐feet wrinkles, forehead wrinkles and the nasolabial fold decreased by 9%, 11% and 5.2%, respectively, compared to baseline (all *P* < 0.05) (Fig. 1).

**Figure 2:**
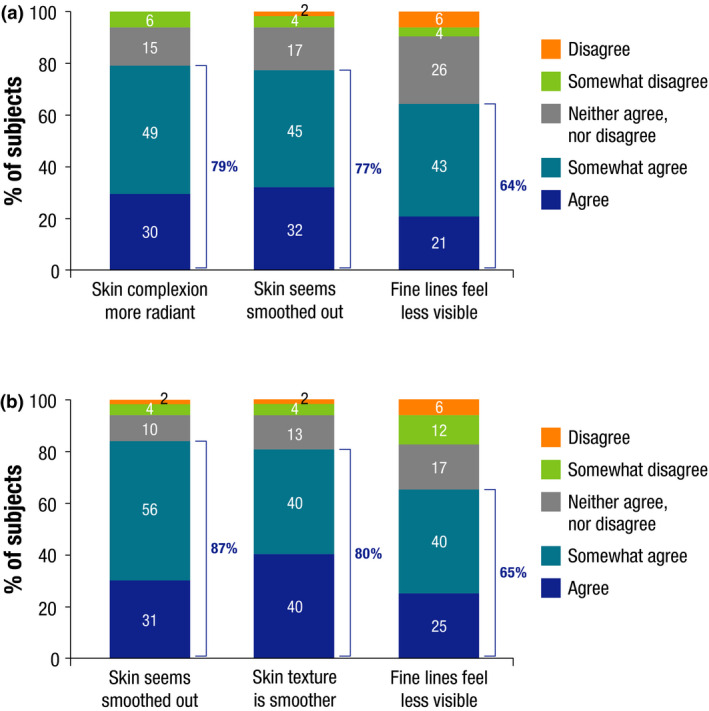
Self‐assessment questionnaire responses at end of study in (a) the clinical scoring study (N = 47) and (b) the instrumental quantification study (N = 51).

Of 47 subjects who completed the self‐assessment questionnaires, the percentage of subjects indicating that after 28 days application of Peptide‐C ampoules their skin complexion was more radiant, their skin seemed smoothed out, and fine lines were less visible were 79%, 77% and 64%, respectively (Fig. 2a).

### Study 3: instrumental quantification on morphology of wrinkles

A total of 53 subjects were enrolled and 52 completed the study as 1 subject discontinued due to an adverse reaction of pruritus on her eyelid (on day 9), which was considered by the investigator as doubtful whether it was related to the investigational product.

All 52 subjects were Caucasian females with a mean age of 49.1 ± 4.5 years old (range: 40–55 years). At baseline, all subjects had crow’s‐feet wrinkles of grade > 2 and < 5 on the photographic scale and 50% of subjects had sensitive skin.

**Figure 3:**
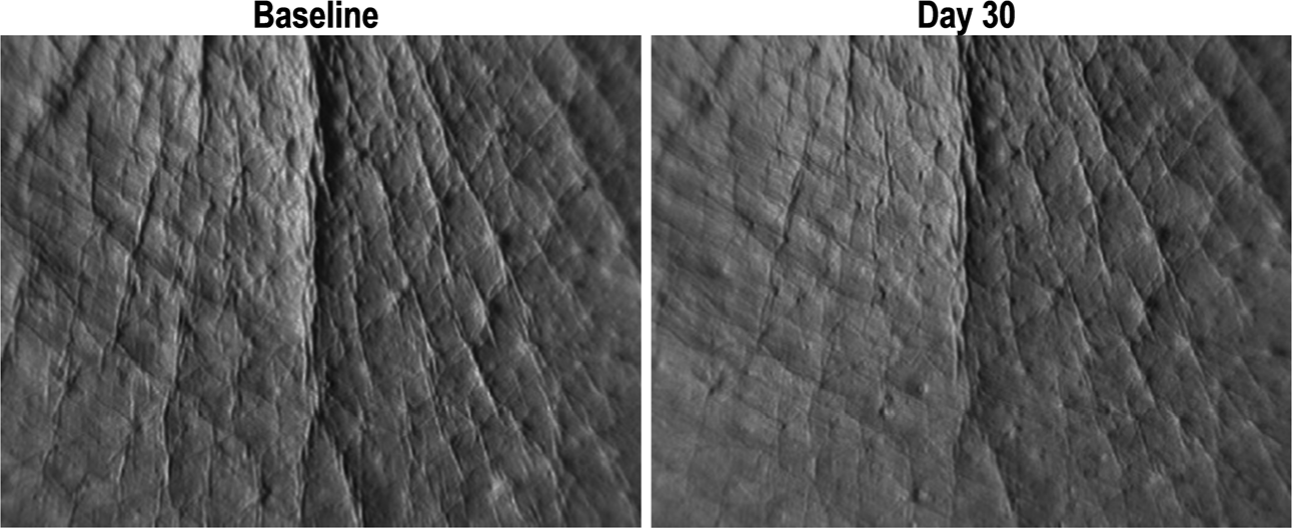
Representative photographs of a median case showing the Quantiride analysis at baseline and on day 30 after 29 days twice‐daily application of Peptide‐C ampoules.

Of 41 subjects completing the instrumental study (Quantirides and Toposurf analysis), 40 subjects had valid prints of crow’s‐feet wrinkles (one subject had a protocol deviation for the baseline print). After 29 days twice‐daily application of Peptide‐C ampoules, the Quantirides results showed a statistically significant decrease of the wrinkles and fine lines total surface, number, total and mean length (Table 1). The number of wrinkles decreased 11.5% (*P* < 0.05). Representative photographs of a median case showing the Quantiride analysis at baseline and the end of the study are shown in Fig. 3. After 29 days of application of Peptide‐C ampoules, the Toposurf analysis showed improvements in the topography of the crow’s‐foot wrinkle print (Table 1). After 29 days of application, the maximal vertical distance between the highest peak and lowest valley decreased 13% compared to baseline (*P* < 0.05).

**Table 1:** Evolution of the crow’s foot wrinkle print between baseline and day 30 (after 29 days of application of Peptide‐C ampoules).

	Mean % evolution at day 30 vs. baseline (N = 40)	*P*‐value
Quantirides evaluation		
Number of wrinkles	−11.5	0.01
Total surface (mm^2^)	−15.7	0.01
Total length (mm)	−13.1	0.01
Mean length (µm)	−4.6	0.032
Toposurf 3D fringe projection analysis		
Maximal vertical distance between the highest peak and the lowest valley (µm)	−13.2	<0.0001
Mean depth of the valleys (µm)	−5.4	0.028

Of 51 subjects who completed the subject self‐assessment analysis, the percentage who agreed that their skin seemed smoothed out, skin texture was smoother and fine lines were less visible, were 87%, 80% and 65%, respectively (Fig. 2b). Overall, 81% of subjects were quite satisfied or very satisfied with the product.

## Discussion

Vitamin C and peptides are widely used in dermocosmetic products, which highlight the need for *in vivo* studies to evaluate whether the various formulations are actually effective in human skin. However, there remains a paucity of well‐designed, *in vivo* clinical studies to substantiate claims [7, 15, 16].

The formulation described here contains pure vitamin C (10%), combined with biopeptides, hyaluronic acid, and Vichy volcanic mineralising water. As vitamin C (ascorbic acid) is sensitive to heat and light, the Peptide‐C formulation is packaged in amber glass ampoules, which contain a daily dose in a minimalist formulation. We evaluated a complete formulation of Peptide‐C topical serum and so we cannot draw conclusions on the mechanism of action of the individual ingredients.

Previous *in vitro* studies on the peptides derived from rice have shown that the optimised peptide structure of an extract rich in di‐ and tripeptides stimulated the proliferation of fibroblasts, stimulating expression of pro‐collagen, collagen VII and fibrillin‐1 [13]. Furthermore, previous clinical studies using instrumental quantification of skin replicas to evaluate *in vivo* the rice extract formulated at 4% in an emulsion have demonstrated an anti‐wrinkle effect of the active ingredient rich in rice di‐ and tripeptides [17].

Previous *in vitro* studies on this formulation demonstrated that Peptide‐C product (5%) has high antioxidant capacity to protect against oxidative stress [18]. Human keratinocyte cell cultures demonstrated that active mix (0.025%) protects against cell damage by reducing oxidative stress, and co‐cultures of human keratinocytes and fibroblasts demonstrated that the active mix (0.01%) increases the neosynthesis of collagen [18].

Dansyl chloride labelling of the stratum corneum and then measuring the time taken for the fluorescence to be eliminated from the skin reflects the rate at which the desquamating stratum corneum has been replaced by the underlying proliferative epidermis. Treatment with the formulation of Peptide‐C ampoules demonstrated a potential to increase the rate of cell exfoliation and renewal of the skin epidermis relative to that of untreated skin, demonstrating its potential for skin rejuvenation.

The investigator clinical scoring study demonstrated that the formulation of Peptide‐C ampoules is effective on the main facial wrinkles, including both horizontal and vertical wrinkles, namely crow’s‐feet wrinkles, forehead wrinkles and nasolabial folds. The instrumental study on the morphology and severity of wrinkles showed that Peptide‐C ampoules reduced the number, surface and length of the crow’s‐foot wrinkle, as measured by Quantirides evaluation. Similarly, measuring the width and depth of the ‘‘peaks’’ and ‘‘valleys’’ of the skin surface by Toposurf 3D fringe projection analysis showed improvements in the topography of the designated crow’s‐foot wrinkle. These results, reflecting a smoothening of the skin surface of the crow’s‐foot wrinkle after 29 days application of Peptide‐C ampoules, were consistent with the investigator clinical scoring data showing improvement in fine wrinkling. Subjective evaluations by the subjects corroborated the clinical and instrumental assessments and, in general, the subjects were satisfied with the treatment. At the end of the studies, the majority of subjects in both studies responded that their skin seemed smoothed out (77% and 87%) and fine lines were less visible (64% and 65% in the respective studies). The main limitations of these studies are that they were not vehicle‐controlled and were of short duration. However, statistically significant differences were consistently observed between Peptide‐C ampoules and baseline in the two open clinical and instrumental studies, as well as between Peptide‐C ampoules and untreated skin in the investigator‐blinded dansyl chloride study.

## Conclusion

This formulation of Peptide‐C topical serum (containing peptides and vitamin C), in innovative packaging allowing a minimalist formula, consistently demonstrated effectiveness and high subject satisfaction for wrinkle reduction and skin rejuvenation.

## References

[1] Flament, F. , Bazin, R. , Laquieze, S. , Rubert, V. , Simonpietri, E. and Piot, B. Effect of the sun on visible clinical signs of aging in Caucasian skin. Clin. Cosmet. Invest. Dermatol. 6, 221–32 (2013). 10.2147/CCID.S44686 PMC3790843 24101874

[2] Wild, C.P. Complementing the genome with an "exposome": the outstanding challenge of environmental exposure measurement in molecular epidemiology. Cancer Epidemiol. Biomarkers Prev. 14, 1847–50 (2005). 16103423 10.1158/1055-9965.EPI-05-0456

[3] Krutmann, J. , Bouloc, A. , Sore, G. , Bernard, B.A. and Passeron, T. The skin aging exposome. J. Dermatol. Sci. 85, 152–61 (2017). 27720464 10.1016/j.jdermsci.2016.09.015

[4] Pullar, J.M. , Carr, A.C. and Vissers, M.C.M. The Roles of Vitamin C in Skin Health. Nutrients. 9, (2017). 10.3390/nu9080866 PMC5579659 28805671

[5] Shibuya, S. , Ozawa, Y. , Toda, T. *et al*. Collagen peptide and vitamin C additively attenuate age‐related skin atrophy in Sod1‐deficient mice. Biosci. Biotechnol. Biochem. 78, 1212–20 (2014). 25229861 10.1080/09168451.2014.915728

[6] Kameyama, K. , Sakai, C. , Kondoh, S. *et al*. Inhibitory effect of magnesium L‐ascorbyl‐2‐phosphate (VC‐PMG) on melanogenesis in vitro and in vivo. J. Am. Acad. Dermatol. 34, 29–33 (1996). 8543691 10.1016/s0190-9622(96)90830-0

[7] Gorouhi, F. and Maibach, H.I. Role of topical peptides in preventing or treating aged skin. Int. J. Cosmet. Sci. 31, 327–45 (2009). 19570099 10.1111/j.1468-2494.2009.00490.x

[8] Schagen, S.K. Topical Peptide Treatments with Effective Anti‐Aging Results. Cosmetics. 4, 1–14 (2017).

[9] Lintner, K. and Peschard, O. Biologically active peptides: from a laboratory bench curiosity to a functional skin care product. Int. J. Cosmet. Sci. 22, 207–18 (2000). 18503476 10.1046/j.1467-2494.2000.00010.x

[10] Katayama, K. , Armendariz‐Borunda, J. , Raghow, R. , Kang, A.H. and Seyer, J.M. A pentapeptide from type I procollagen promotes extracellular matrix production. J. Biol. Chem. 268, 9941–4 (1993). 8486721

[11] Lintner, K. Promoting production in the extracellular matrix without compromising barrier. Cutis. 70(6 Suppl), 13–6; discussion 21–3 (2002). 12498533

[12] Pauly, G. , Contet‐Audonneau, J.‐L. , Moussou, P. *et al*. Small proteoglycans in the skin: new targets in the fight against skin aging. Int. J. Cosmet. Sci. 31, 154. (2009).

[13] Jouandeaud, M. Di‐ and Tripeptides: A new approach to skin nutrition. Personal Care Magazine. 1–4 (2002).

[14] Pinnell, S.R. , Yang, H. , Omar, M. *et al*. Topical L‐ascorbic acid: percutaneous absorption studies. Dermatol Surg. 27, 137–42 (2001). 11207686 10.1046/j.1524-4725.2001.00264.x

[15] Michalek, I.M. , Lelen‐Kaminska, K. and Caetano Dos Santos, F.L. Peptides stimulating synthesis of extracellular matrix used in anti‐ageing cosmetics: Are they clinically tested? A systematic review of the literature. Australas. J. Dermatol. 60, e267–e71 (2019). 30941744 10.1111/ajd.13036

[16] Lee, C.M. Fifty years of research and development of cosmeceuticals: a contemporary review. J. Cosmet. Dermatol. 15, 527–39 (2016). 27496663 10.1111/jocd.12261

[17] Guzman, A.‐I. , Boudier, D. , Guichard, N. , Gofflo, S. and Closs, B. Natural peptides: “active”nutrients for deficient skins. Peptides naturels : des nutriments « actifs » pour les peaux carencées. Expression cosmétique Guide of cosmetic ingredients. December 2015:266–70. (2015).

[18] Martín‐Martínez, A.S.‐M.N. , Martínez‐Casanova, D. , Abarquero‐Cerezo, M. , Herranz‐López, M. , Barrajón‐Catalán, E. and Matabuena‐Yzaguirre, M. High global anti‐oxidant protection (RMS) and stimulation of the collagen synthesis of new anti‐age product containing an optimized active‐mix. IMCAS World Congress poster presentation; Paris, France; 2020 30 January – 1 February.

